# Supplementary material for: Stress, Anxiety, and Depression Levels among University Students: Three Years from the Beginning of the Pandemic
Source: Clin Pract. 2023 Apr 27;13(3):596–609. doi: 10.3390/clinpract13030054 (PMC10204477; doi:10.3390/clinpract13030054)
Supplement: Supplementary file 1 [file clinpract-13-00054-s001.zip › clinpract-2338939-supplementary.pdf]

Table S1. Demographic characteristics of the students during the 3rd year of completing the questionnaire.

**Characteristics**	***Participants on 2020 (%)****	***Participants on 2021 (%)****	**Participants on 2022 (%)**
**Age**			
18-25	*1719 (74.0)*	*2369 (75.0)*	1137 (76.0)
> = 26	*603 (26.0)*	*791 (25.0)*	360 (24.0)
**Gender**			
Female	*1694 (73.0)*	*2205 (69.8)*	999 (66.8)
Male	*628 (27.0)*	*955 (30.2)*	498 (33.2)
**Marital Status**			
Unmarried/Single	*1971 (84.9)*	*2781 (88.0)*	1362 (91.0)
Married/Other	*351 (15.1)*	*379 (12.0)*	135 (9.0)
**Live with**			
Alone	*340 (14.6)*	*983 (31.1)*	457 (30.5)
1 person	*477 (20.5)*	*799 (25.3)*	382 (25.5)
2 or more people	*1505 (64.9)*	*1378 (43.6)*	658 (44.0)
**Vaccinated**			
Yes	-	*2602 (82.3)*	1248 (83.4)
No	-	*558 (17.7)*	249 (16.6)
**Total**	*2322 (100.0)*	*3160 (100.0)*	1497 (100.0)

Kavvadas et al.* **[4]

Table S2. Questions about Covid-19 during the two years.

**Students' Characteristics**	***Participants on 2020 (%)****	***Participants on 2021 (%)****	**Participants on 2022 (%)**
**Know patient with COVID-19 diagnosis**			
Yes	*2033 (87.6)*	*2984 (94.4)*	1480 (98.8)
No	*289 (12.4)*	*176 (5.6)*	18 (1.2)
SUM	*2322 (100.0)*	*3160 (100.0)*	1497 (100.0)
**If YES, Reported COVID-19 symptoms**			
No Symptoms	*117 (5.8)*	*87 (2.9)*	17 (1.1)
Mild	*896 (44.1)*	*1007 (33.7)*	497 (33.6)
Moderate	*647 (31.8)*	*1202 (40.3)*	735 (49.7)
Severe	*254 (12.4)*	*420 (14.1)*	130 (8.8)
Death	*119 (5.9)*	*268 (9.0)*	101 (6.8)
**Total of those reported YES**	*2033 (100.0)*	*2984 (100.0)*	1480 (100.0)

**Kavvadas et al. [4]*

Table S3. Students' mental health characteristics and social burden due to the pandemic.

Characteristics	*Participants on 2020 (%)**	*Participants on 2021 (%)**	**Participants on 2022 (%)**
**Previous psychological or psychiatric treatment**			
Yes	*439 (18.9)*	*796 (25.2)*	348 (23.2)
No	*1883 (81.1)*	*2364 (74.8)*	1149 (76.8)
**Current psychological or psychiatric treatment**			
Yes	*159 (6.8)*	*458 (14.5)*	170 (11.4)
No	*2163 (93.2)*	*2702 (85.5)*	1327 (88.6)
**Current intake of psychoactive medication**			
Yes	*48 (2.1)*	*122 (3.9)*	57 (3.8)
No	*2274 (97.9)*	*3038 (96.1)*	1440 (96.2)
**Total**	*2322 (100.0)*	*3160 (100.0)*	1497 (100.0)
******Kavvadas et al. [4]*			

Table S4. University status of students.

**Students**	*Participants on 2020 (%)**	*Participants on 2021 (%)**	**Participants on 2022 (%)**
**Educational Rank**			
Undergraduate Students	*1724 (81.7)*	*2394 (82.1)*	1114 (74.4)
Master Students	*286 (13.6)*	*385 (13.2)*	291 (19.4)
PhD Students	*100 (4.7)*	*137 (4.7)*	92 (6.2)
**Total of Students**	*2110 (100.0)*	*2916 (100.0)*	1497 (100.0)
******Kavvadas et al. [4]*			

Table S5. Vaccination against Covid-19 infection and correlations (November, 2022)

**Vaccinated:**	**YES**	**NO**	**TOTAL**	**p-values (chi-squared)**
**Sex**				
Female	827	167	994	.870
Male	416	82	498	
**Educational Rank**				
Undergraduate Students	911	203	1114	**.0082**
Master Students	259	32	291	
PhD Students	80	12	92	

Table S6. Concern about impending lockdown in correlation to gender (November, 2022).

**Worries about lockdown:**	**NOT AT ALL**	**A LITTLE**	**MUCH**	**VERY MUCH**	**SUM**	**p-values (chi-squared)**
Female	343	439	145	72	999	**<.00001**
Male	241	167	53	37	498	
**Total**	584	606	198	109	1497	
